# Pulsed processing by cold plasma, applied to industrial emission control

**DOI:** 10.3389/fchem.2024.1386055

**Published:** 2024-06-07

**Authors:** E. J. M. Van Heesch, T. Huiskamp, K. Yan, F. J. C. M. Beckers, H. W. M. Smulders, G. J. J. Winands, R. H. P. Lemmens, P. P. M. Blom, S. Davalos Segura, W. F. L. M. Hoeben, S. V. B. Van Paasen, J. J. Van Oorschot, A. G. A. Bonkestoter, M. L. J. Van Den Brand, M. Hennink, R. W. J. Smulders, A. J. M. Pemen, P. C. T. Van Der Laan

**Affiliations:** ^1^ Electrical Engineering Department, Eindhoven University of Technology, Eindhoven, Netherlands; ^2^ Institute of Industrial Ecology and Environment, Zhejiang University, Hangzhou, China; ^3^ Solybright BV, Heesch, Netherlands; ^4^ HMVT, Ede, Netherlands; ^5^ Antea Group, Oosterhout, Netherlands

**Keywords:** cold plasma, nanosecond pulses, pulsed processing, plasma processing, VOC, emission control, Lambert function

## Abstract

A promising pollution control technology is cold plasma driven chemical processing. The plasma is a pulsed electric gas discharge inside a near atmospheric-pressure-temperature reactor. The system is energized by a continuous stream of very short high-voltage pulses. The exhaust gas to be treated flows through the reactor. The methods applied involve the development of robust cold plasma systems, industrial applications and measuring technologies. Tests of the systems were performed at many industrial sites and involved control of airborne VOC (volatile organic compound) and odor. Electrical, chemical and odor measuring data were collected with state-of-the-art methods. To explain the test data an approximate solution of global reaction kinetics of pulsed plasma chemistry was developed. It involves the Lambert function and, for convenience, a simple approximation of it. The latter shows that the amount of removal, in good approximation, is a function of a single variable. This variable is electric plasma power divided by gas flow divided by input concentration. In the results sections we show that in some cases up to 99% of volatile pollution can be removed at an acceptable energy requirement. In the final sections we look into future efficiency enhancements by implementation of (sub)nanosecond pulsed plasma and solid state high-voltage technology and by integration with catalyst technology.

## 1 Introduction

Pollution control is an ever-increasing concern. Government policies can adapt to new technologies in three aspects of pollution: source, measurement, control. A control technology that has been emerging since the 1980s is cold plasma driven pollution abatement (Goldman et al., 1985; Mizuno et al., 1986; Masuda and Nakao, 1986; 1988; van Heesch, et al., 1989; 2008; 2012; Chang et al., 1991; Rosocha et al., 1993; 2000; Yan et al., 2001; Winands et al., 2006; Hoeben et al., 2012; Beckers et al., 2013; Huiskamp et al., 2014; 2017; 2019; 2020). The plasma is a pulsed electric gas discharge. It appears as a cloud of electric mini sparks contained in a reactor at atmospheric pressure. This cloud is being energized by a stream of very short high-voltage pulses. The air to be treated flows through the reactor. The processes run continuously over a long period of time (hours to days or months). However, as mentioned, the high voltage is not continuous but pulsed. The pulses are very short (5 ns - 5 microsecond) and are repeated at high rate (100 Hz–10 kHz). Pulsing the plasma over a long time means that chemical processing behavior is pulsed as well as quasi continuous; actually, the process is a near infinite row of short duration, high repetition rate processing steps. To be able to realize this continuous operation, systems with robust high-voltage power source and reactor have been developed. They can operate over long periods of time (weeks to months).

Important challenges within this technology area are raising the levels of performance in the areas: removal efficiency, peak pulse power, pulse width, pulse repetition rate, average power, energy efficiency, electromagnetic compatibility (EMC), overall reliability and resilience to dust and condensing water.

The main purpose of this paper is to present the principle of cold plasma processing, two industrial plasma processing systems and results of case studies performed with these systems, a deeper look at plasma processing kinetics, and finally a look into the future of plasma processing.

### 1.1 Cold plasma processing

The cold plasma is also called non-thermal plasma (NTP). It can, for instance, be produced by nanosecond duration (5 ns - 100 ns) high-voltage (HV) pulses applied to a concentric HV wire–grounded cylinder electrode geometry. The metal cylinder carries the flow of air. The pulses are short to prevent full electric breakdown. Alternatively, we can apply a dielectric barrier between these electrodes. This configuration allows much longer duration pulses, microsecond to millisecond range. Non-thermal means that the plasma electrons gain relatively high energies (are hot) as they are effectively accelerated during the very short and very high electric field phase. The bulk of the gas, the heavy particles (molecules, ions, excited states and fragments) remain cold (low thermal energy) since they are not much accelerated by the short duration electric field. The accelerated energetic electrons are the actors that initiate the chemical reactions. They have energies in the range of the bonding energies of the molecules. In addition to excited states and ions, electron-air impact creates reactive oxygen and nitrogen species, further abbreviated as RONS; examples are the hydroxyl radical (OH), ozone (O_3_), hydrogen peroxide (H_2_O_2_) and transient and stable H_x_N_y_O_z_ species. These radicals are responsible for the conversion or abatement of chemical compounds in a pulsed plasma reactor. The radicals are not stable in air. For instance, O-radicals combine with O_2_ as ozone. Ozone is rather stable but is much less effective than the OH and O radicals. The nanosecond plasma is more powerful in generating the energetic electrons, and thus radicals, than is the microsecond plasma. Cold pulsed plasmas for chemical processing were reported already in the 1980s, e.g., in (Goldman et al., 1985; Masuda et al., 1986; 1988; Mizuno et al., 1986). High pulsed currents in streamer-plasma reactors were first documented in 1989 (van Heesch et al., 1989).

## 2 Methods applied

The applied pulsed plasma technology relies basically on the combination of a pulsed power source and a plasma reactor at atmospheric conditions. The pulsed power source delivers a flow of electrical energy pulses to the reactor. Each pulse is ca. 5–100 ns wide and has a power level that peaks at levels up to hundreds of Mega Watt. These pulses are repeated at a rate of 10–5,000 pulses per second.

The pulsed power source takes its energy from the grid and delivers it to the plasma in the reactor at an efficiency of up to around 80%. The plasma delivers this energy to the electrons with an efficiency between ca. 1%–50% (van Heesch et al., 2008).

The electrons initiate the chemical process. This process is a chemical conversion from reactants into products. The final result is conversion. This conversion can be expressed as the concentration of reactants converted, divided by the concentration of reactants on input.

The conversion will be compared with the electrical energy needed to perform this conversion. This electrical energy is expressed as an energy density. This energy density is defined as electrical energy divided by treated volume. This is the same as the electrical power into the reactor divided by volumetric gas flow into the reactor. It is the crucial parameter for pulsed plasma processing. Species particle concentration for reactants and products are the other important parameters.

**FIGURE 1:**
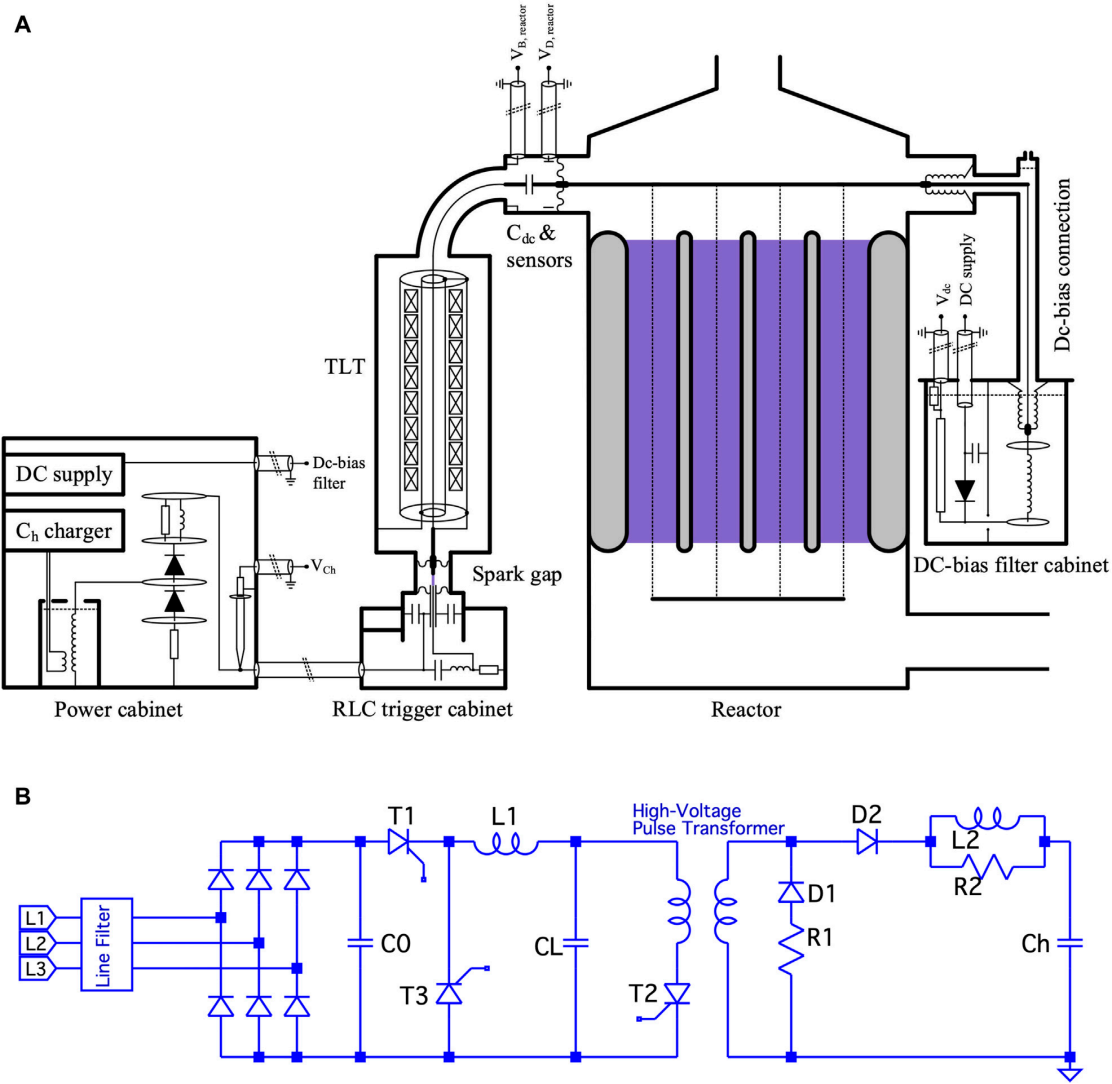
**(A)** Layout of the nanosecond cold plasma system (Beckers, 2015). **(B)** Basic layout of the circuit called “Ch charger” in panel A is given by the left part of the circuit in panel **(B)**. The functionality is described in Beckers (2015).

**FIGURE 2:**
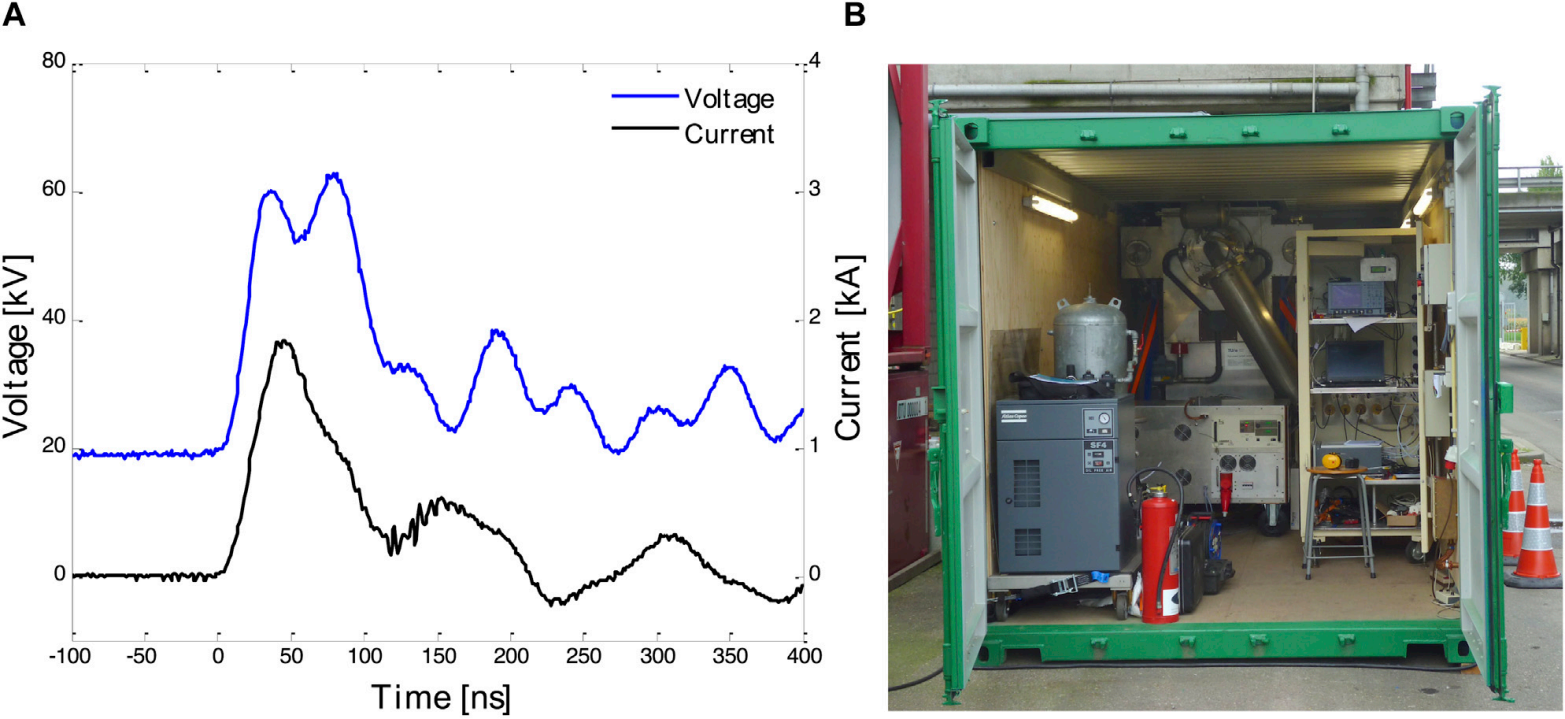
**(A)** Typical voltage and current traces for the plasma reactor of the nanosecond system. **(B)** The container with the nanosecond system. More information in Beckers (2015).

**FIGURE 3:**
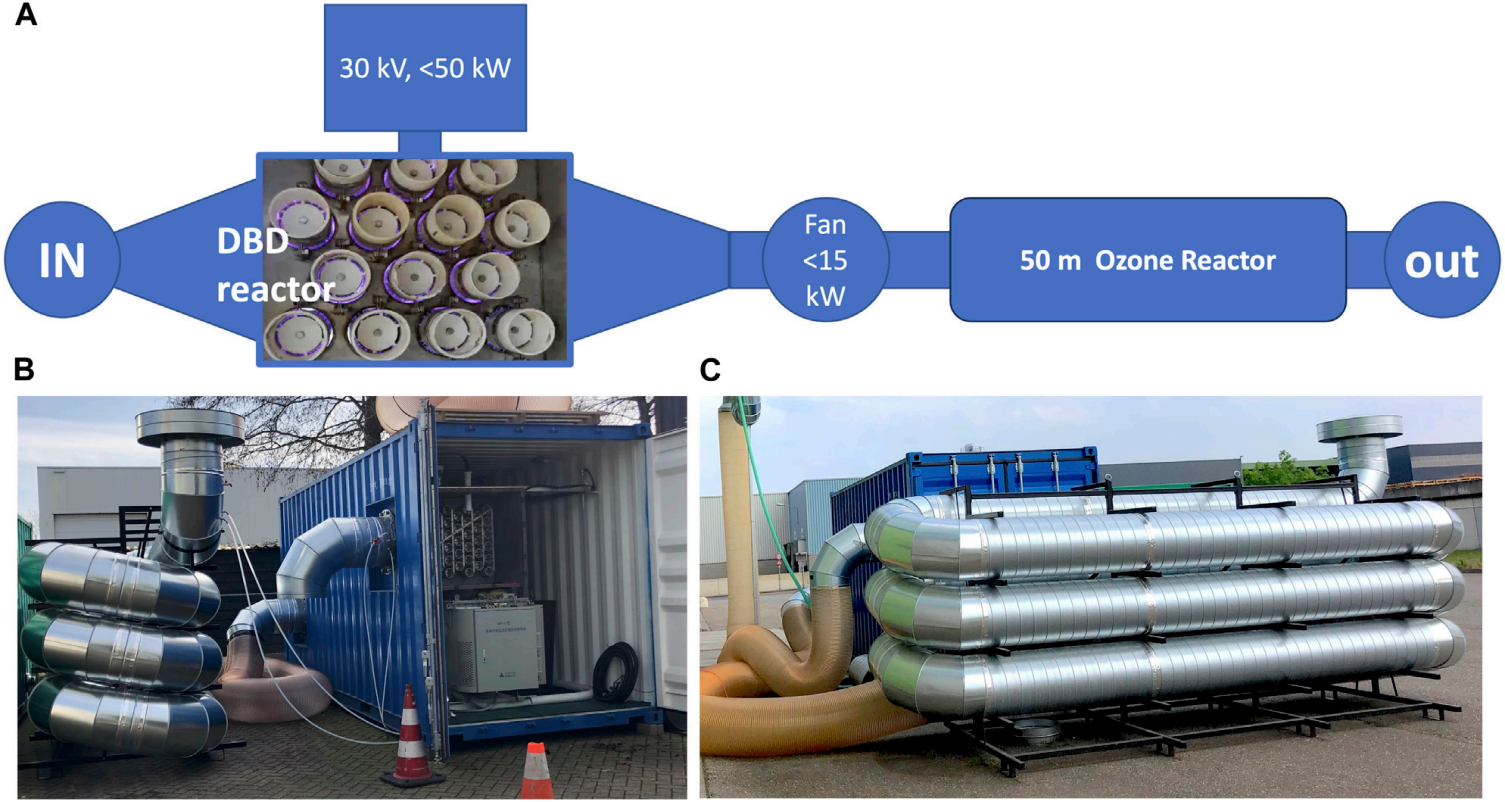
**(A)** Layout of the microsecond cold plasma system. **(B,C)** Photos of the container housing the microsecond system and of the ozone reactor.

**FIGURE 4:**
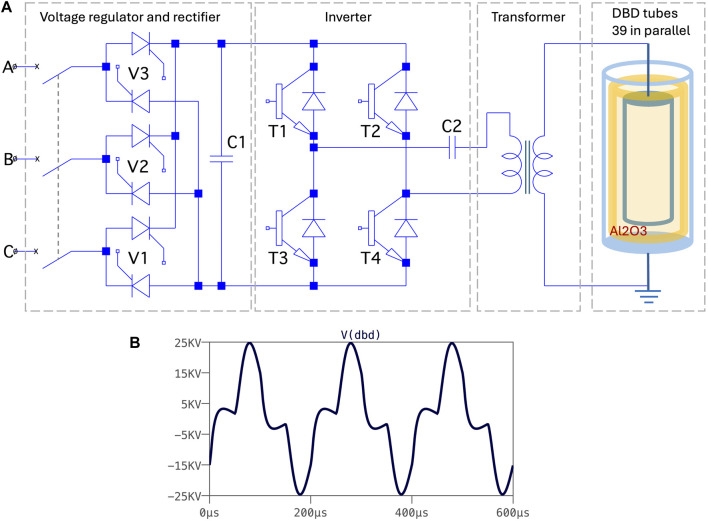
**(A)** Electric layout (simplified) of the microsecond cold plasma system. More information in Li et al. (2018). **(B)** Typical voltage traces at the terminals of the DBD plasma reactor of the microsecond system. We obtained this result by applying circuit simulation software (LTspice) for fitting the calculated response of circuit to the measured behavior.

A 5 kW wire-cylinder based nanosecond plasma machine was applied. It is described in detail in, e.g., Beckers 2013. It was developed at Eindhoven University of Technology. The basic layout and overview are given in Figures 1A, B and Figures 2A, B. We also applied a 20 kW DBD-based (dielectric barrier discharge) microsecond plasma machine. It was developed by colleagues in China in a cooperation project with NL. Its basic layout and overview are given in Figures 3A–C and in Figures 4A, B. The voltage pulses applied to the nanosecond device differs considerably from the pulses delivered to the microsecond device. The differences are illustrated by Figure 2B, 4B.

The 5 kW and the 20 kW device were mounted in 20-ft freight containers for housing, easy transport and on-site installation. They are equipped with fully automated control. The control covers: reactor flow, energy flow, safety, electrical measurements, flow measurements, and full pulsed-power control. Both systems can be remotely controlled via a protected internet connection.

The microsecond plasma machine was equipped with a post-processing device. A 50-m-long tube, added to the output of the plasma machine serves as plug flow reactor to convert residual ozone. During the tubes transit time, further oxidation occurs of VOCs by ozone.

Layout and pictures of the device are shown in Figures 3A–C.

During one of our test campaigns, we made a comparison with an industrial RONS injector device. This injector technology is commercially available from several manufacturers. Basically, it concerns a millisecond plasma machine, 50 Hz repetition rate, dielectric barrier discharge. It is housed in a reactor chamber separate from the exhaust process flow. A very clean air flow is fed into the device and the output flow is injected into the exhaust flow, the stack, of the industrial process. Chemically active species from plasmas (radicals), except ozone, have short lifetimes (100 microsecond or less). The relatively long transit time from plasma production chamber of the injector device to stack will reduce the effectiveness of the active species. Ozone will easily survive the transit time but for O and OH radicals it will be difficult.

## 3 Pulsed processing behavior

The input concentration will be denoted by *C0*, the output concentration by *C*. The conversion will be denoted by *X* where *X* equals (*C0-C)/C0*. The energy density will be denoted by *E* and equals *P/F*, where *P* is the power into the reactor and *F* the volumetric flow into the reactor. The unit we use for the energy density is Wh/m^3^ (1 Wh/m^3^ = 3.6 J per Liter). For concentrations, we use ppm, parts per million. The behavior of the conversion process can be approximated by Eq. 1 (van Heesch et al., 2012). We derived this equation as an extension of the groundbreaking work by Yan et al. (2001). Eq. 1 is valid in the approximation of *a.N* > 1, where *a* is the combined plasma volume, average during one pulse, divided by the reactor volume and *N* is the number of pulses applied during reactor transit time. The value of *a* cannot be well established but can be assumed to be very small, like 10^−4^. That means the N must be large.

The entire approach is based on:

1. Global chemical kinetics developed in (van Paasen et al., 1997; Yan et al., 2001)
2. Multiple pulsed plasma volumes in which reactive species are generated by a high-voltage pulse
3. The sum of transient plasma volumes created during the reactor transit should be larger than the reactor volume (a.N > 1)
4. Reactive species induced oxidative degradation of target compounds
5. Termination of reactive species
6. Remixing of the plasma volumes into the reactor after each pulse

The resulting behavior can be given as:

$$\gamma .\ln\left(1-X\right)-X.C0+k.E=0$$ (1)

where γ and *k* are characteristic numbers for a specific process. γ is a characteristic value of concentration (in ppm). Variable *k* (in ppm.m^3^/Wh) is the radical production per unit of energy density, as given in the already mentioned publication (van Heesch et al., 2012). Both parameters are constants for a given process and can be evaluated in two ways: from the theoretical analysis and from a data fit. However, to evaluate the theoretical expressions for these parameters, given in van Heesch et al., 2012, detailed knowledge of rate constants would be needed. In the present case, we performed a least-squares fit on the data to obtain values for γ and k.

Equation 1 is implicit in the unknown *X*. It can be found that the solution involves the application of the Lambert function *W(z).* This function is defined as the inverse of *f(z), where f(z) = z.exp(z).* We only use the positive part of the principal branch of *W*, denoted by (*W*_*0*_ *),* and apply real arguments. We used Mathematica from Wolfram (Weisstein, 2002) to obtain the explicit solution of Eq. 1:

$$X=\frac{C0-C}{C0}=1-\gamma .\frac{W_{0}\left(Y\right)}{C0}$$ (2)

where

$$Y=\left(\frac{C0}{\gamma }\right).e^{\left(\frac{C0}{\gamma }-k.\frac{E}{\gamma }\right)}$$ (3)

**FIGURE 5:**
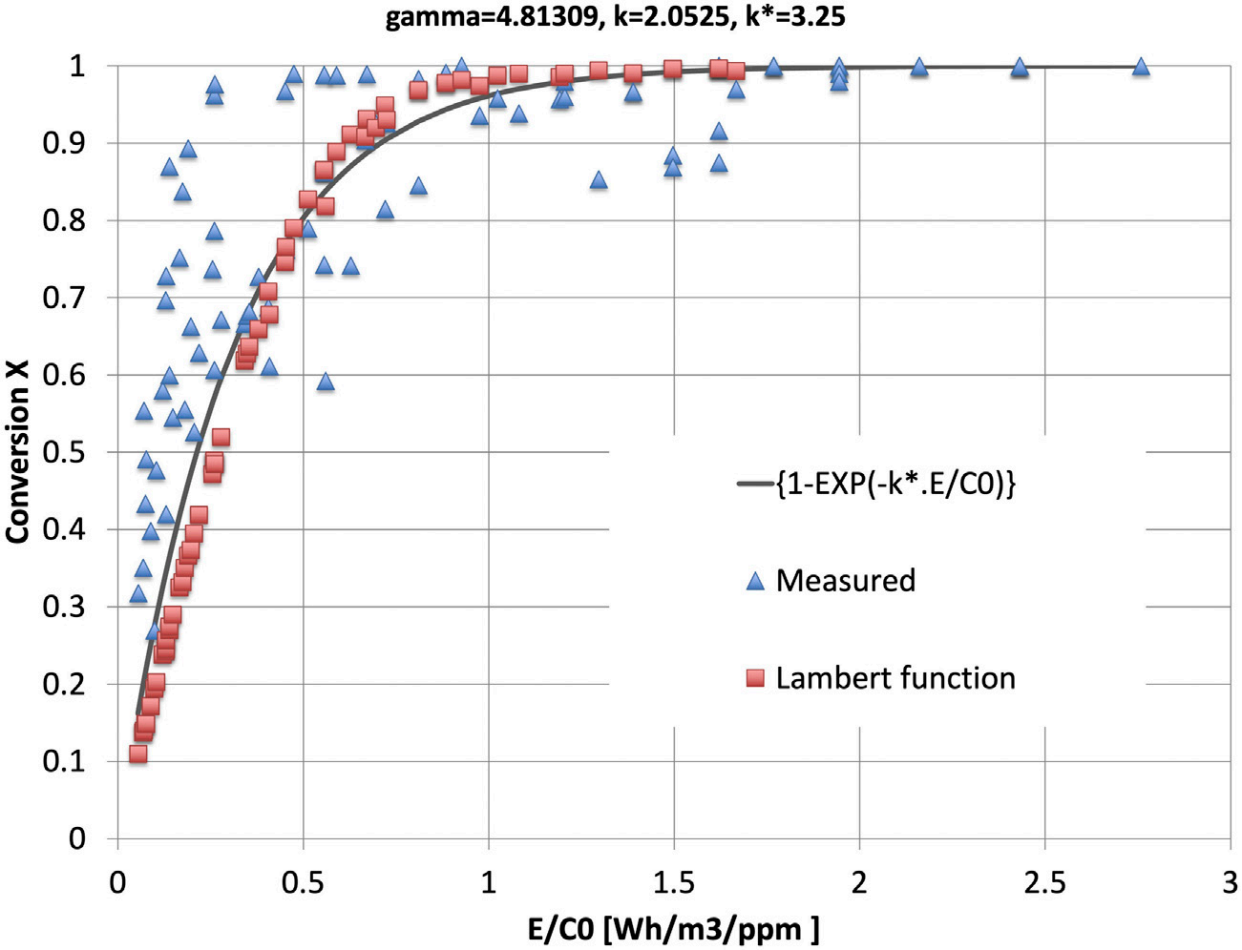
2D graph of conversion as a function of the parameter E/C0 . The triangles are the 82 measured data points collected during 3 days of on-site testing. The squares are the values according to the solution Eq. 2 (Lambert function) of Eq. 1. The smooth curved line is according to the approximation in Eq. 4.

Figure 5 shows the behavior of the value of the conversion X, as a function of the parameter *E/C0*. Three data sets have been plotted: the measured data, the model according to the Lambert function (Model 1) and a simplified expression according to an exponential function of k.E/C0 (Model 2):

$$X={1-e}^{\left(-k^{*}.\frac{E}{C0}\right)}$$ (4)

Equation 4 was already given in 1986 by Masuda as based on the best fit to his experimental data (Masuda and Nakao, 1986).

**FIGURE 6:**
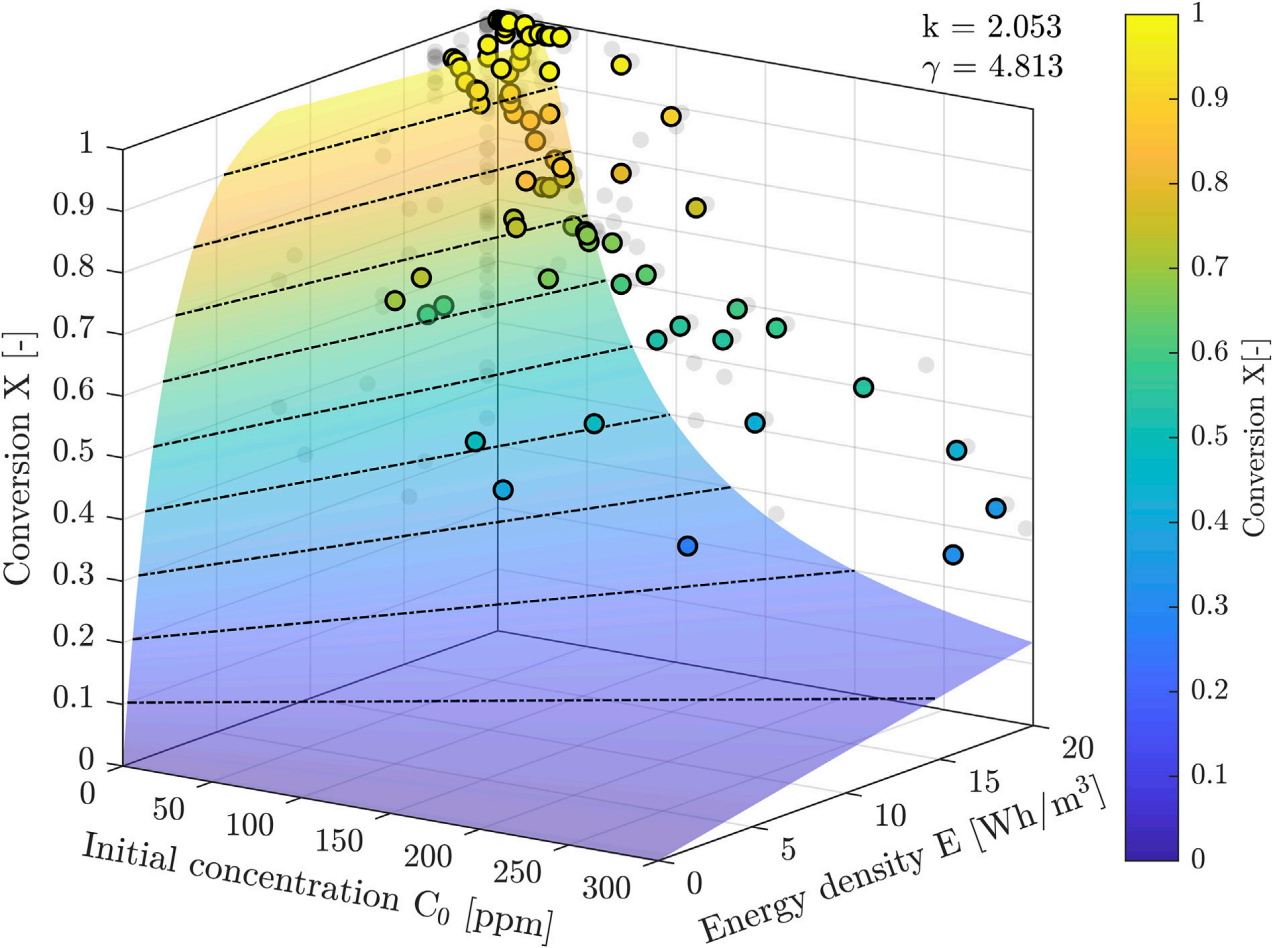
3D surface as the implicit solution of Eq. 1. The measuring data are superimposed. The dashed black lines are lines of constant X. The X-E-planes to the left (small C0) show model 3 and the X-E planes to the right (large C0) show model 4.

**FIGURE 7:**
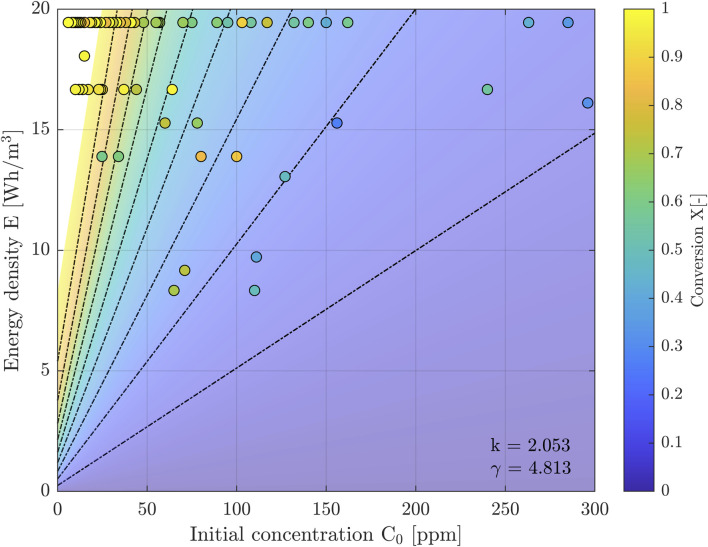
2D graph that is the projection of the surface plot in Figure 6. Dashed black lines are lines of constant X.

The parameters k and γ in Eqs 2, 3 were fitted (least squares, linear) to our measured data to obtain γ = 4.813 ppm and *k* = 2.053 ppm m^3^/Wh. Figure 6 shows the 3D plot of the solution of Eq. 1, fitted to the measuring data. Figure 7 is the 2D projection of the 3D surface of Figure 6.

The best fit between the data points and Eq. 4 is obtained for a slightly different value than the one for *k* in the calculations to obtain Figure 6. We denote the related new parameter by *k* ^*^, and its best fit is *k* ^*^ = 3.25 ppm m^3^/Wh.

We conclude that the test data fit the model according to the Lambert function with some scatter. The scatter in the data points according to the Lambert function also stems from the fact that it is a function of independently *E* and *C0* and not exactly a function of the *x*-axis variable *E/C0*. The function in Eq. 4, is a very simple function of the single variable *E/C0*, and quite well follows the model according to the more complicated Lambert function. The function in Eq. 4 now is proposed as a generalization of existing models (Rosocha et al., 1993; 2000; van Paasen et al., 1997; Smulders et al., 1998; Yan et al., 2001):

$$\text{Model }3\;X=1-e^{-E/SIE}$$ (5)

$$\text{Model }4\;X=k^{*}.E/C0$$ (6)

where *SIE* (specific energy density, sometimes denoted as beta in literature) is the engineering constant for a specific process, used in literature. *SIE* is the specific input energy, defined as the energy density needed to obtain a 1/e reduction of the pollutant concentration. We can notice the following: Eq. 4 has a power series expansion that for small arguments ε, ε = (*k* ^*^ *.E/C0*), that simplifies to Eq. 6.

Furthermore, it can be noticed that Eq. 5 is the analogue of Eq. 4 for *SIE* = *C0/k* ^*^. This latter observation shows that the model based on an *SIE* value can only be used if *C0* is constant during the test, and that the actual *SIE* value depends on the value of *C0* of the specific test.

Moreover, these findings show that the model according to Eq. 4 combines both former models given by Eqs 5, 6.

The 3D graph of Figure 6 was made to show the global behavior of the implicit solution of Eq. 1. The axes ranges were chosen in accordance with the ranges during the described measuring campaign. The measuring data are superimposed. The figure also clearly shows the models 3 and 4 of Eq. 5 (small C0 X-E-plane) and 6 (large C0 X-E-plane).

## 4 Case studies with the 5 kW and the 20 kW systems

### 4.1 Odor control at an animal feed production plant, case 1

Testing was performed during a project for plasma assisted odor reduction at an animal feed plant. The described 20 kW microsecond DBD system was fed with a flow that was branched off from the main flow. The DBD system has the earlier mentioned rest-ozone reactor as the permanent post-processing unit.

The industry plant was equipped with a plasma injector system fed into the main flow, far behind the branch-off location for the DBD system. This injector device was running permanently.

**FIGURE 8:**
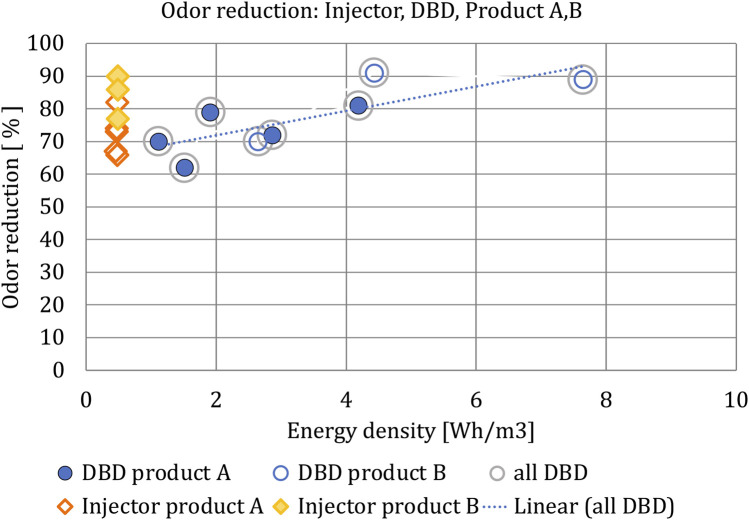
Odor reductions of up to 91% were achieved with the DBD machine. The injector machine showed odor reductions of up to 90%. Two animal feed products **(A, B)** have been tested. The product with the highest protein content **(B)** has the highest odor reduction. For both **(A,B)**, an energy density setting for the DBD system significantly higher than that of the plasma injector machine does not lead to significantly more odor reduction by the DBD system.

The performance of both machines on the process output was monitored during 3 days while the plant product spectrum was varied a few times. Odor emissions were measured according to certified procedures (EN 13725) by a certified lab (Odournet). This lab also monitored the VOC emissions using GC-TOF-MS (gas chromatograph–time of flight–mass spectrometer) screening. Simultaneously we monitored the energy densities for both machines. The results are summarized in Table 1 and Figure 8.

**TABLE 1:** Results of GCMS and odor measurements at an animal feed production facility. We compared the concentration in the input of the two considered plasma systems with the concentrations in the output of the DBD-Ozone reactor system and with the concentration in the output far behind the plasma injector system.

units are µg/m3 or ou_E_/m3	Product A		
	in	out (DBD)	out (injector)
average GCMS sum full	1,572	3,000	3,729
average GCMS sum > odor threshold	189	263	725
average odor units	12,010	3,400	3,098

	Product B		
	in	out (DBD)	out (injector)
average GCMS sum full	21,584	17,903	9,826
average GCMS sum > odor threshold	5,302	3,873	2,485
average odor units	20,760	1,985	1,728

The project proved that the investigated cold plasma DBD machine functions well and reliably during long times of operation, even under harsh conditions, at an industrial plant. We were able to perform extensive measurements and analysis of the performance of a 20 kW DBD machine for the industrial odor emissions of an animal feed plant. Odor reductions of up to 91% were achieved with the DBD machine. The plasma injector machine showed odor reductions of up to 90%. Two animal feed products (A and B) have been tested. The product with the highest protein content (B) has the highest odor reduction. For product A there is no clear correlation between the reduction of total VOC (volatile organic compounds) concentration and the reduction of the odor concentration. For product B there is a slightly positive correlation between total VOC reduction and the odor reduction. For both A and B, an energy density setting for the DBD system significantly higher than that of the plasma injector machine does not lead to more odor reduction by the DBD system. A higher energy density does lead to more VOC reduction.

The (fine) dust emissions from the plant, and its interaction within the complex of particles-VOCs-SVOCs-oxidation-water, (SVOC, semi-volatile organic compound) have consequences for the performance, the measurements, and the analysis. Fine dust removal following the production process should be at a higher rate than present to prevent contamination of the DBD machine. Contrary, an injector system does not pollute from fine dust because the injector plasma source is not in contact with the process air.

VOC clustering into aerosols by ozone and radicals can in theory also bind the odorous VOCs. This possible mechanism could in theory mask odorants. The water vapor condensation also leads to interactions with VOCs. This system of particles-VOCs-oxidation-water can mask or can cause odors and can also mask and cause VOCs.

Ad hoc solutions have been found for the nuisance caused by condensation of water by adding water conduits and by temperature management of the DBD machine. In a full-scale application, the DBD machine must be kept at a temperature near or above the dewpoint of the exhaust gas of the plant. The ideal location of the full-scale DBD machine is therefore in line with the chimney.

### 4.2 Polycyclic aromatic hydrocarbon conversion and postprocessing performance, case 2

The 20 kW microsecond system in combination with the ozone reactor placed behind the plasma reactor, has been tested for emissions from a hot mix asphalt manufacturing facility and for effectiveness of the rest-ozone reactor. As in Section 4.1, the ozone reactor is the post processing device that employs the rest ozone from the microsecond plasma system. The emissions being tested contain polycyclic aromatic hydrocarbons (PAH). In the present case, Naphthalene is the major constituent of the PAH.

We measured total VOC content with a three-channel FID (flame ionization detector) setup.

Channel 1 measures the VOC content in the flow from the stack of the asphalt plant. Channel 2 measures the VOCs after plasma process. Channel 3 measures the output gas from the ozone reactor.

**FIGURE 9:**
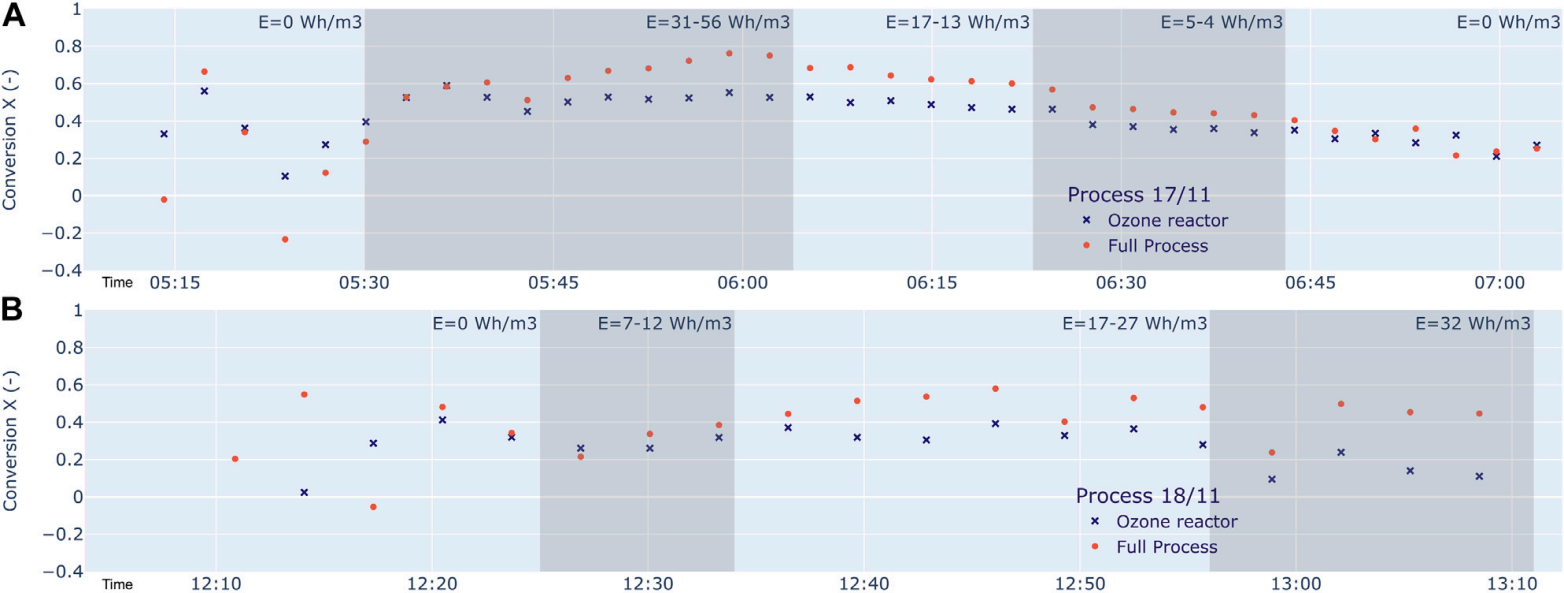
Time records of the conversion X for the full process (DBD plus Ozone reactor) and for the Ozone reactor alone for two different Asphalt products. **(A)** The process 17/11: We see a conversion larger than 40% under wide spectrum of parameters, and in some cases between 70% and 80%. The rest-ozone reactor is very effective. The post-processing conversion of this device alone is up to 55%. **(B)** The process 18/11: We see a total conversion of up to 59%. The post-processing device alone converts up to 40%. It turns out that the product 18/11 is more difficult to convert. For cases **(A,B)**, the total conversion X and the share in it by the DBD-reactor increase with energy density E.

The results are shown in Figure 9 and can be described as follows.

During the time from start and end of a tests we notice a time delay effect between settings of the energy density and the response seen in the conversion. In parallel we notice a background conversion that possibly can be attributed to capture of PAH by aerosol formation around mist droplets. We should keep in mind these uncertainties in the following summary:

At an energy density of 17 Wh/m3 we see a conversion of 69% on total VOC for process 17/11. At an energy density of 56 Wh/m3 we see a conversion of up to 78%. On all measurements we see a conversion larger than 40% under wide spectrum of parameters, and in some cases between 70% and 80%. The rest-ozone reactor is very effective. The post-processing conversion of this device alone is up to 55%. For the process 18/11 we see a total conversion of up to 59%. The post-processing device alone converts up to 40%. It turns out that the product 18/11 is more difficult to convert.

During operation of the microsecond plasma machine we encountered a problem due to water vapor condensation if reactors and piping cool down below the dewpoint temperature of the exhaust gas of the plant. To partially solve this problem, we used a heating device. Maybe or in combination, a demister should be used in future as a supporting solution.

### 4.3 VOC control at a food processing plant, case 3

We performed a test at an industrial site. The 5 kW ns plasma machine was used. The industrial emission concerns an exhaust air flow that is contaminated with volatile organic compounds (VOC), being mainly triglycerides. We treated a partial outflow of the system: 180–400 m^3^ per hour. The power from the pulsed power source into the plasma is 0–4 kW. The input VOC concentration is 6–296 ppm (parts per million). We monitored the VOC concentrations simultaneously on input to the reactor and on output, in front of the carbon filter. The carbon filter was used to reduce the ozone emission from the plasma into the stack. The measuring devices were two similar FID (flame ionization detector) analyzers. The composition of the waste gas flow is very complex. We apply integral FID detection and apply the propane equivalent to obtain ppm (parts per million). In special cases, where knowledge about the actual composition is required, a differential FID technique is needed. In that case the detection is preceded by chromatographic separation (Hoeben et al., 2012). Simultaneously, we measure the energy density using the detection system that is an integral part of the pulsed power source (Beckers et al., 2013).

For a range of input parameters, the total conversion was measured and documented. We collected 82 data points, for *C0,* the input concentration, and for the accompanying output concentration *CN*. The results have been summarized in Figures 5–7.

### 4.4 Odor control at a manure processing facility for fertilizer production, case 4

Testing was performed for odors/VOCs from processing manure into fertilizers. We applied the 5 kW ns plasma machine. The rest-ozone reactor was not available. A small post processing device was connected the output of the machine. It is an activated carbon filter of 0.45 m^3^. Odor was monitored by a certified laboratory. Total VOC content was measured by the Mulitrae-Lite PID (photo-ionization detector) device.

The tests were done during a 1.5-h (run1) and a 4-h run (run2). The results are summarized in Table 2.

**TABLE 2:** Results of tests with the nanosecond system on odor and VOC conversion at a facility that processes manure into fertilizer.

Run	Flow	Energy density	Total VOC in	Total VOC out	Average odor in	Average odor out	VOC conversion	Odor conversion
	m^3^/h	Wh/m^3^	ppm	ppm	ou_E_/m^3^	ou_E_/m^3^	%	%
1	400	2.8	8.7	2	153,994	619	77.0	99.6
2	410	2.8	7.2	0.2	13,121	911	97.2	92.7

The tests prove that odor reduction is effective for a manure processing plant by application of a nanosecond plasma machine in combination with a small carbon filter. The energy cost is acceptable. Extrapolating the energy density to the full exhaust flow of 100,000 m^3^/h of the plant, we calculate a power need of 280 kW for the plant. This number fits in the range of the other power needs of the plant, like fans and heating.

### 4.5 VOC control at manure processing for compost production, case 5

The nanosecond plasma system was applied at a manure processing facility. The product of the process is compost. We applied the nanosecond cold plasma system (Winands et al., 2006). The VOC samples were collected by Pra-Odournet BV and analyzed by GC-MS.

Various combinations of conventional processing and cold plasma processing were investigated.

It turned out that the certified odor measurements only partially reflect the results of the chemical measurements.

More in detail:

The case acid scrubber nearly off in combination with cold plasma at its high mode (3.2 Wh/m^3^) has a good performance. These results are summarized in Table 3. The calculated odor conversion is 95%, measured odor conversion 43%, H_2_S conversion 95% and VOC conversion 87%.

**TABLE 3:** Results of tests with the nanosecond system on odor and VOC conversion at a facility that processes manure into compost: The case acid scrubber nearly off in combination with cold plasma at its high mode (3.2 Wh/m^3^).

	Input [μg/m^3^]	Output [μg/m^3^]	Conversion [%]
Aromatic CH’s	395	10	97
Cyclic CH’s	66	0	100
Aliphatic CH’s	271	13	95
Alcohol	787	44	94
Esters	0	263	-
Ketone	3,881	273	93
Aldehydes	946	570	40
Chlorinated components	200	0	100
Organic Sulphur	2,363	2	100
Furane	66	10	84
Terpenes	13	0	100
Total	8,987	1,185	87
H_2_S [ppm]	10.1	0.5	95
Odor calculated [ou_E_/m^3^]	4,197	208	95
Odor certified lab [ou_E_/m^3^]	135,398	77,854	43

The case acid scrubber fully on and cold plasma at its medium mode (2 Wh/m3) has a rather mixed performance. The calculated odor conversion is 14%, measured odor conversion 83%, H_2_S conversion 95%, but the VOC conversion has turned into VOC production.

See Table 4.

**TABLE 4:** Results of tests with the nanosecond system on odor and VOC conversion at a facility that processes manure into compost: The case acid scrubber fully on and cold plasma at its medium mode (2 Wh/m3).

	Input [μg/m^3^]	Output [μg/m^3^]	Conversion [%]
Aromatic CH’s	152	101	34
Cyclic CH’s	0	68	-
Aliphatic CH’s	65	430	−566
Alcohol	3,796	1,357	64
Organic acids	0	100	-
Ketone	9,510	5,242	45
Aldehydes	1,305	35,930	−2,654
Chlorinated components	443	135	70
Organic Sulphur	3,348	101	97
Furane	219	0	100
Terpenes	27	0	100
Esters	504	35	93
Other	10	0	100
Total	19,729	43,787	−122
H_2_S [ppm]	10.1	0.5	95
Odor calculated, [ou_E_/m^3^]	5,244	4,489	14
Odor certified lab [ou_E_/m^3^]	160,289	26,816	83

## 5 Methods in development: very short pulses for more efficient nanosecond cold plasma processing

In the drive towards industrialization of the pulsed plasma technology, two aspects are important: energy efficiency and solid-state technology. Both these topics will be addressed in this section, where we look towards future implementations of pulsed plasma systems for industrial air cleaning.

### 5.1 Increasing energy efficiency

**FIGURE 10:**
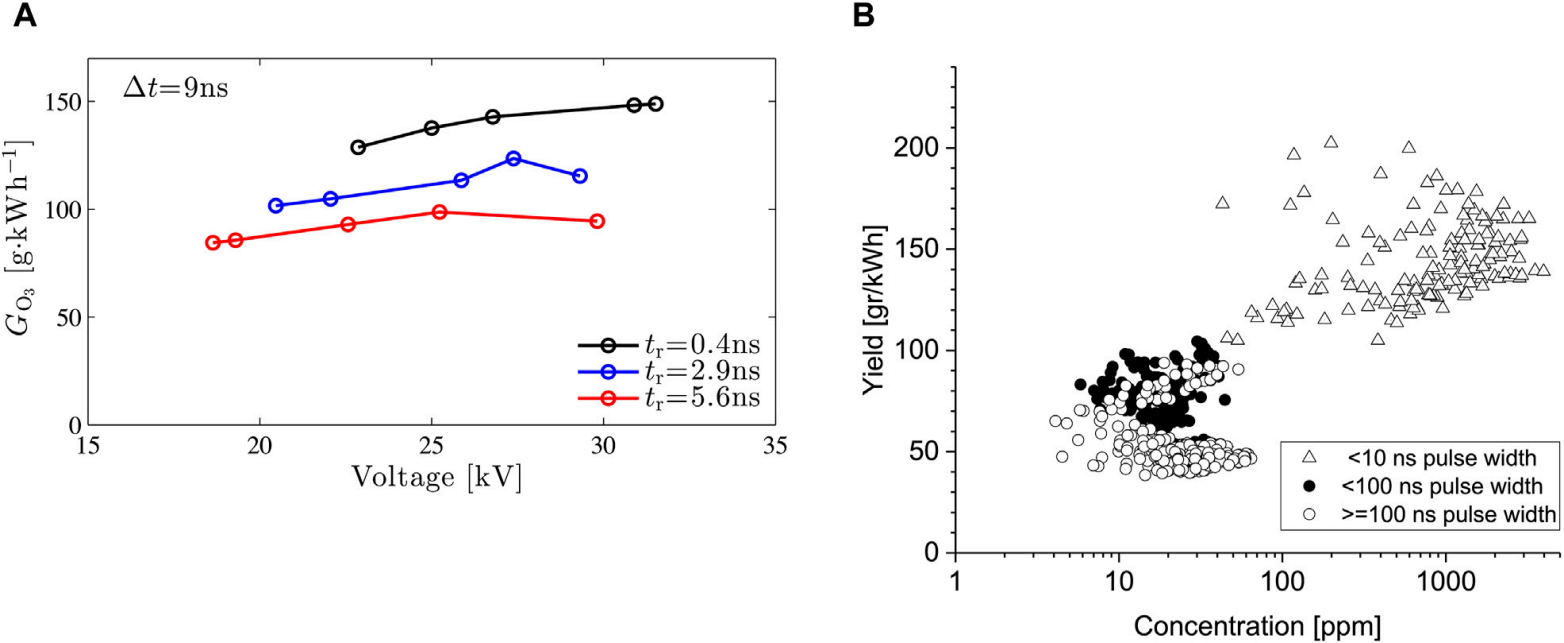
**(A)** Ozone production efficiency as a function of the applied voltage and rise time in a 50-mm diameter, 1-m long, coaxial pulsed plasma produced by the sub-nanosecond pulse source. **(B)** Overview of ozone generation yields and concentrations for different experiments done in Eindhoven. The <10 ns results are those performed with the sub-nanosecond pulse source. The other results are those of systems such as shown in, e.g., Figures 1, 2.

In literature, we often see that using short (nanosecond) high-voltage pulses result in high efficiencies of different plasma processes, such as ozone production, NO removal, etc. More specifically, research has shown that very short high-voltage pulses achieve the highest efficiencies (van Heesch et al., 2008; Matsumoto et al., 2010; Ono et al., 2011; Huiskamp et al., 2017; Post et al., 2024). We specifically designed a sub-nanosecond rise time pulse source to investigate the limits of energy efficiencies. It is a repetitive (up to several kHz), flexible, single-line nanosecond pulse source with a rise time of several hundreds of picoseconds. To obtain the short rise time we implemented single-line pulse source with a transmission line and an oil flushed spark gap. We can vary the pulse duration from 0.5 to 10 ns and can adjust the amplitude in the range ± 0–50 kV (Huiskamp et al., 2014). With it, we showed that, while short pulses result in high efficiencies, it is mainly the rise time of the pulses that has a large effect (if the pulses are short enough): the shorter the rise time, the higher the energy efficiency (where energy efficiency is defined as the amount of radicals produced in the plasma as a function of the energy used by the plasma) (Huiskamp et al., 2017). An example is shown in Figure 10A.

An overview of results from pulsed corona systems from Eindhoven is shown in Figure 10B. The results show that while the industrial nanosecond system (from Figures 1, 2) performs very well, it is outperformed by the sub-nanosecond pulse source. However, the sub-nanosecond pulse source is very much a lab prototype using an oil spark gap, unfit for industrial operation. Looking to the future, the aim is to achieve the results of the sub-nanosecond pulse source, but then with a pulsed power system that lends itself for commercialization. One such a system would be a solid-state system.

### 5.2 Solid-state flexible waveform generation for plasma processing

As described in the previous subsection, the energy efficiency of the plasma can be enhanced by operating it with very short pulses. Ideally, such a system uses solid-state (semiconductor) components (such as the system in Figure 4), while maintaining the high plasma efficiency. Therefore, we need a pulse source topology capable of generating such short pulses (<10 ns rise time and duration) with semiconductors, while still being able to maintain full control over the application by ensuring we can flexibly change pulse parameters such as pulse duration, amplitude, shape, etc. Unfortunately, this rules out most of the known solid-state nanosecond pulse generators (Huiskamp, 2020). Therefore, we introduced a new topology.

**FIGURE 11:**
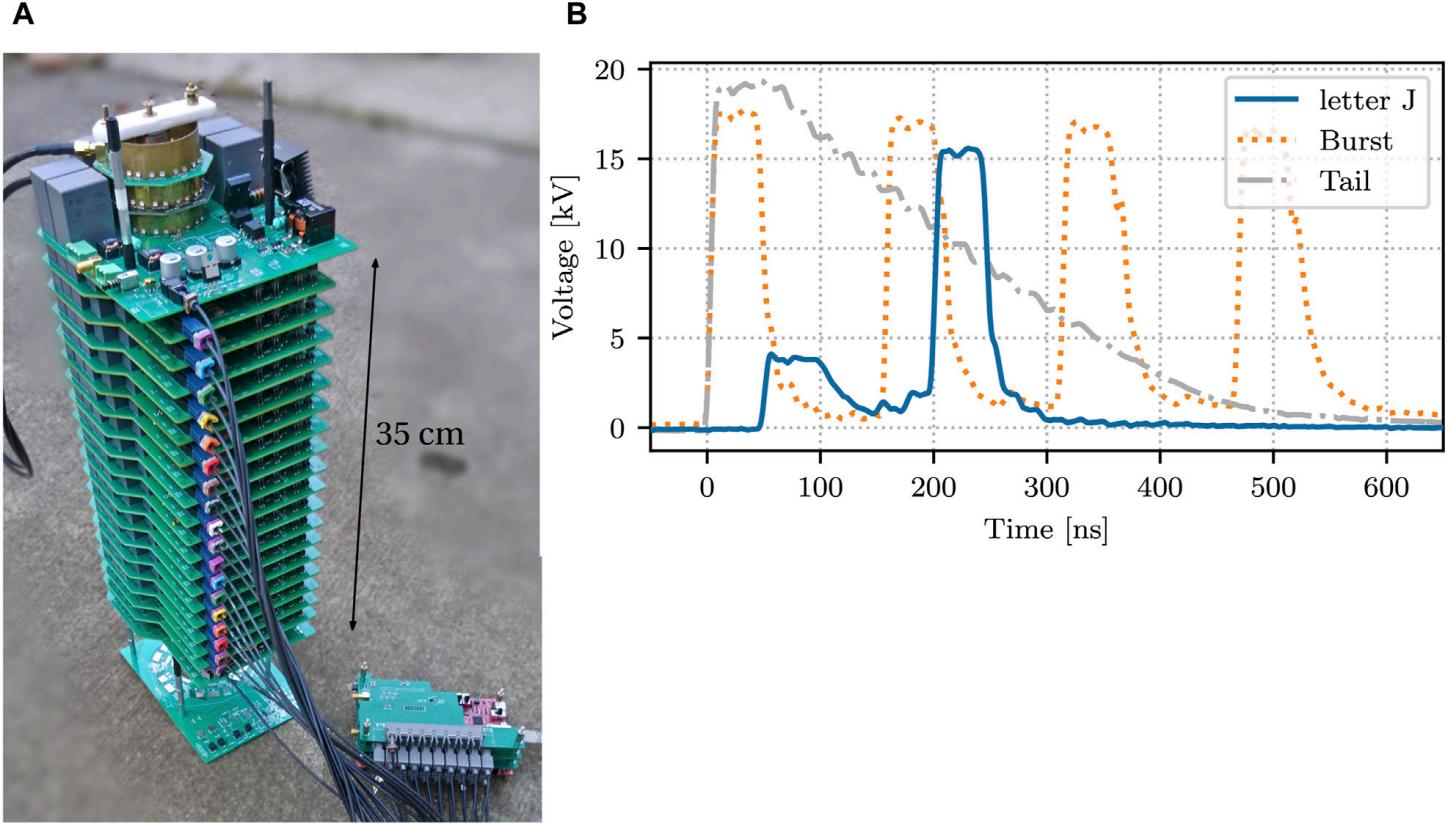
**(A)** The 20-kV solid-state IMG. **(B)** Some example waveforms (van Oorschot and Huiskamp, 2023).

Based on the topology of the Impedance-Matched Marx Generator (IMG) presented in 2017 by researchers from Sandia (and others) (Stygar et al., 2017), we created a solid-state IMG using MOSFET switches (Huiskamp and van Oorschot, 2019; van Oorschot and Huiskamp, 2023). The advantage of using the IMG topology is that by using transmission lines to transmit the pulses from the Marx stages the rise time of the pulses can be maintained at the output waveform (when carefully impedance-matched). By designing the Marx stages very compactly and using fast semiconductor components, adjustable pulses with rise times of just several nanoseconds are feasible with this topology. We developed a 20-kV prototype, capable of 10-kHz repetition rate, 20-ns pulse duration, 5-ns rise time with a programmable waveform into loads of 50–200 Ω. The pulse source and some example waveforms are shown in Figures 11A, B and we will use it in future research to optimize pulsed power plasma processing.

## 6 Conclusion and discussion

In this paper we presented the principle of cold plasma processing, two industrial plasma processing systems and results of case studies performed with these systems, a deeper look at plasma processing kinetics, and finally a look into the future of plasma processing.

The developed apparatus for VOC abatement performed very well. Tests conducted on VOC and odor removal at industrial sites in Netherlands proved that in some cases, up to 99% reduction is achievable. For the PAH Naphthalene, being a hard to convert VOC, a conversion of 75% could be obtained, which is a promising result. To summarize the different treatment methods and results, the key parameters of the reported methods and the existing injection method are given in Table 5.

**TABLE 5:** Ratings, indicative, of the reported treatment methods. Estimates are on basis of collected data. Case numbers refer to the case numbers in the section headings. (cost items are for a large installation, 100.000 m^3^/h exhaust flow, and at an electric energy cost of 0.1 € per kWh).

Application ⇓	Parameter	Cold plasma (+postprocessing)		Injection
		DBD microsec	Wire-cylinder nanosec	
**Odor** (animal feed **case 1**)	energy setting	1 Wh/m^3^		0.5 Wh/m^3^
	running cost	10 €/h		5 €/h
	installation cost	400 k€		400 k€
	removal	++++		++++
	must operate > dewpoint	yes		no
**VOC** (asphalt **case 2**)	energy setting	20 Wh/m^3^		
	running cost	200 €/h		
	installation cost	8,000 k€		
	removal	+++		
	must operate > dewpoint	yes		
**VOC** (food processing **case 3**)	energy setting		10 Wh/m^3^	
	running cost		100 €/h	
	installation cost		8,000 k€	
	removal		+++	
	must operate > dewpoint		yes	
**Odor + VOC** (manure processing **case 4**)	energy setting		2–3 Wh/m^3^	
	running cost		25 €/h	
	installation cost		2000 k€	
	removal		++++	
	must operate > dewpoint		yes	
**Odor + VOC** (manure processing **case 5**)	energy setting		3 Wh/m^3^	ca. 0.5–2 Wh/m^3^
	running cost		30 €/h	ca. 10 €/h
	installation cost		2,400 k€	ca. 800 k€
	removal		+++	--
	must operate > dewpoint		no	no

The global processing behavior has been analyzed theoretically and experimentally. It turns out that, in both these aspects, the pulsed plasma process behaves according to the Lambert function. We showed that existing processing models have a common ground in the Lambert function.

The argument of the Lambert function in the case of plasma processing, is a combination of input pollutant density C0 and energy density E. A workable approximation for this Lambert function is an exponential function of the argument E/C0. This functionality was already given by Masuda in 1986 as a fit to his experimental data.

The future of cold plasma processing needs to develop towards increasing efficiency and robustness. This enhancement will be facilitated by development and introduction of fully solid-state high-voltage pulsed power (short rise times, short pulses, high pulse repetition rates) and by the integration of the (sub)nanosecond cold plasma with cold catalyst technology. Research and applications that integrate the strengths of these two technologies are gaining much interest in recent years. Synergy is found in several crosslinked areas of cold plasma and catalysis. As already pointed out by (Cheng et al., 2021; Kim et al., 2021) the development of pulsed power for short nanosecond pulses with kHz range repetition rate is needed to unlock the magical key of vibrational energy in plasma catalysis. Table 6 is a summary of successful combinations of nanosecond pulsed plasma and catalysis (Kim et al., 2016; 2021; Wang et al., 2018; Ronda-Lloret et al., 2020; Cheng et al., 2021; Shao et al., 2022; Zeng et al., 2023; Chen et al., 2024).

**TABLE 6:** Synergy in four types of application areas.

Application area ⇒ reactor _⇓_	Catalyst preparation	Catalyst regeneration	VOC oxidation	Chemical reforming
Cold Plasma	**+**	**+**	**+**	**+**
Catalyst + Cold Plasma			**++**	**++**

## Data Availability

The raw data supporting the conclusion of this article will be made available by the authors, without undue reservation.
